# Does respiratory syncytial virus lower respiratory illness in early life cause recurrent wheeze of early childhood and asthma? Critical review of the evidence and guidance for future studies from a World Health Organization-sponsored meeting

**DOI:** 10.1016/j.vaccine.2020.01.020

**Published:** 2020-03-04

**Authors:** Amanda J. Driscoll, S. Hasan Arshad, Louis Bont, Steven M. Brunwasser, Thomas Cherian, Janet A. Englund, Deshayne B. Fell, Laura L. Hammitt, Tina V. Hartert, Bruce L. Innis, Ruth A. Karron, Gayle E. Langley, E. Kim Mulholland, Patrick K. Munywoki, Harish Nair, Justin R. Ortiz, David A. Savitz, Nienke M. Scheltema, Eric A.F. Simões, Peter G. Smith, Fred Were, Heather J. Zar, Daniel R. Feikin

**Affiliations:** a Center for Vaccine Development and Global Health, University of Maryland School of Medicine, 685 W. Baltimore St, Suite 480, Baltimore, MD, USA; b The David Hide Asthma and Allergy Research Centre, St. Mary’s Hospital, Newport PO30 5TG, Isle of Wight, UK; c Clinical and Experimental Sciences, Faculty of Medicine, University of Southampton, Southampton General Hospital, Tremona Road, Southampton SO16 6YD, UK; d The ReSViNET Foundation, Zeist, the Netherlands; e Department of Pediatric Infectious Diseases, Wilhelmina Children’s Hospital, University Medical Centre Utrecht, Lundlaan 6, Utrecht, the Netherlands; f Department of Translational Immunology, Wilhelmina Children’s Hospital, University Medical Centre Utrecht, Lundlaan 6, Utrecht, the Netherlands; g Center for Asthma Research, Allergy, Pulmonary & Critical Care Medicine, Vanderbilt University School of Medicine, 2525 West End Ave, Suite 450, Nashville, TN 37203, USA; h MM Global Health Consulting, Chemin Maurice Ravel 11C, 1290 Versoix, Switzerland; i Seattle Children’s Hospital, 4800 Sand Point Way NE Seattle, WA 98105, USA; j Department of Pediatrics, University of Washington, 1959 NE Pacific St, Seattle, WA 98195, USA; k School of Epidemiology and Public Health, University of Ottawa, Children’s Hospital of Eastern Ontario (CHEO) Research Institute, 401 Smyth Road, CPCR, Room L-1154, Ottawa, Ontario K1H 8L1, Canada; l Department of International Health, Johns Hopkins Bloomberg School of Public Health, 615 N. Wolfe St, Baltimore, MD 21205, USA; m Center for Vaccine Innovation and Access, PATH, 455 Massachusetts Avenue NW, Suite 1000, WA, DC 20001, USA; n Center for Immunization Research, Johns Hopkins Bloomberg School of Public Health, 624 N. Broadway, Suite 217, Baltimore, MD 21205, USA; o Division of Viral Diseases, National Center for Immunization and Respiratory Diseases, US Centers for Disease Control and Prevention, 1600 Clifton Rd, Atlanta, GA 30329, USA; p Murdoch Children’s Research Institute, Flemington Rd, Parkville, VIC 3052, Australia; q Department of Paediatrics, University of Melbourne, Flemington Rd, Parkville, VIC 3052, Australia; r Epidemiology and Population Health, London School of Hygiene and Tropical Medicine, Keppel St, Bloomsbury, London WC1E 7HT, UK; s Division of Global Health Protection, US Centers for Disease Control and Prevention, PO Box 606-00621, Nairobi, Kenya; t Centre for Global Health Research, Usher Institute, University of Edinburgh, Medical School, Teviot Place, Edinburgh EH8 9AG, Scotland, United Kingdom; u Department of Epidemiology, Brown University School of Public Health, Providence, RI 02903, USA; v Department of Pediatrics, Section of Infectious Diseases, University of Colorado School of Medicine, and Children's Hospital Colorado 13123 E. 16th Ave, B065, Aurora, CO 80045, USA; w Department of Epidemiology, Center for Global Health Colorado School of Public Health, 13001 E 17th Pl B119, Aurora, CO 80045, USA; x Tropical Epidemiology Group, London School of Hygiene and Tropical Medicine, Keppel St, Bloomsbury, London WC1E 7HT, UK; y Department of Pediatrics and Child Health, University of Nairobi, P.O. Box 30197, GPO, Nairobi, Kenya; z Department of Paediatrics and Child Health, Red Cross War Memorial Children’s Hospital, Cape Town, South Africa; aa SA-Medical Research Council Unit on Child and Adolescent Health, University of Cape Town, 5th Floor ICH Building, Klipfontein Road, Cape Town, South Africa; ab Department of Immunizations, Vaccines and Biologicals, World Health Organization, 20 Avenue Appia, Geneva, Switzerland

**Keywords:** Respiratory syncytial virus, Wheeze, Asthma, Vaccine, Monoclonal antibody

## Abstract

Respiratory syncytial virus (RSV) is a leading cause of lower respiratory tract infection (LRTI) and hospitalization in infants and children globally. Many observational studies have found an association between RSV LRTI in early life and subsequent respiratory morbidity, including recurrent wheeze of early childhood (RWEC) and asthma. Conversely, two randomized placebo-controlled trials of efficacious anti-RSV monoclonal antibodies (mAbs) in heterogenous infant populations found no difference in physician-diagnosed RWEC or asthma by treatment group. If a causal association exists and RSV vaccines and mAbs can prevent a substantial fraction of RWEC/asthma, the full public health value of these interventions would markedly increase. The primary alternative interpretation of the observational data is that RSV LRTI in early life is a marker of an underlying predisposition for the development of RWEC and asthma. If this is the case, RSV vaccines and mAbs would not necessarily be expected to impact these outcomes. To evaluate whether the available evidence supports a causal association between RSV LRTI and RWEC/asthma and to provide guidance for future studies, the World Health Organization convened a meeting of subject matter experts on February 12–13, 2019 in Geneva, Switzerland. After discussing relevant background information and reviewing the current epidemiologic evidence, the group determined that: (i) the evidence is inconclusive in establishing a *causal* association between RSV LRTI and RWEC/asthma, (ii) the evidence does not establish that RSV mAbs (and, by extension, future vaccines) will have a substantial effect on these outcomes and (iii) regardless of the association with long-term childhood respiratory morbidity, severe acute RSV disease in young children poses a substantial public health burden and should continue to be the primary consideration for policy-setting bodies deliberating on RSV vaccine and mAb recommendations. Nonetheless, the group recognized the public health importance of resolving this question and suggested good practice guidelines for future studies.

## 1 Background and meeting objectives

Respiratory syncytial virus (RSV) is a leading cause of lower respiratory tract infection (LRTI) and hospitalization in children globally, causing an estimated 33.1 million LRTI episodes, 3.2 million hospitalizations, and 118,000 deaths in 2015 [1]. An estimated 45% of all hospitalizations and deaths are in infants less than 6 months of age, with 99% of global RSV mortality occurring outside of North America and Europe. The only licensed monoclonal antibody (mAb) to prevent RSV LRTI (Synagis®, palivizumab) is recommended only in high-risk infants (e.g. preterm or with certain co-morbidities) and is cost prohibitive for low and middle-income countries (LMICs). There are no licensed vaccines for RSV; however, several candidate products (e.g., vaccines and mAbs) are in clinical development [2].

A long-standing question is whether RSV LRTI in early life causes subsequent recurrent wheeze of early childhood (RWEC) and asthma. The current evidence supporting a causal association between RSV and RWEC/asthma is mixed. Understanding whether prevention of RSV LRTI can lead to reductions in rates of RWEC and asthma will contribute important information to policy decisions regarding RSV vaccines and mAbs.

To shed light on this important question, the World Health Organization (WHO) undertook three activities. The first comprised an analysis of the sample size required to estimate the potential impact of RSV prevention by vaccines or mAbs on the subsequent development of RWEC in RCTs [3]. The second was a systematic review and *meta*-analysis that will be reported separately. Third was a convening of subject matter experts on February 12–13, 2019 in Geneva, Switzerland (Agenda and Participants in Appendix A). The objectives of the meeting were: (i) to evaluate the strength of the current evidence for a causal association between early life RSV LRTI and subsequent RWEC/asthma, (ii) to evaluate the evidence that future RSV vaccines/mAbs can reduce rates of RWEC/asthma, and (iii) to provide methodological guidance for future studies. This report summarizes the meeting.

## 2 Epidemiology of RSV LRTI

Epidemiological studies have shown that more than half of children experience their first RSV infection in the first 12 months of life and almost all will have had an infection by two years of age [4]. Involvement of the lower airways occurs in 15–50% of children with primary RSV infection, with 45% of LRTI occurring in the first 6 months of life [1], [5]. Although children born preterm, with low birth weight, chronic lung disease, congenital heart disease, or immunosuppression have increased risk of severe disease, most children with RSV LRTI were born full-term and have no underlying illnesses [6], [7]. RSV LRTI usually corresponds to a clinical diagnosis of bronchiolitis or pneumonia and is differentiated from RSV upper respiratory tract infection by lower chest wall indrawing, tachypnea, diffuse rhonchi, or wheezing [8]. Wheezing associated with the acute RSV LRTI episode can persist for up to 4 weeks (median 12 days) [9]. The case-fatality ratio for RSV LRTI is low (<1%) if a child receives supportive care in a timely manner, but can be as high as 9% in low-income countries [1].

## 3 Epidemiology of RWEC and childhood asthma

Wheeze, which can refer to both a clinical sign and a reported symptom, is an intrathoracic sound and a sign of airflow limitation [10]. A large proportion of young children experience viral-associated recurrent wheezing, a highly heterogenous condition that is not always indicative of asthma [11]. Asthma represents a disease spectrum with multiple phenotypes and may present with respiratory signs and symptoms including wheeze. A clinical diagnosis of asthma in older children and adults requires a history of symptom patterns and evidence of variable expiratory airflow limitation, which can be assessed by different lung function testing methods [11]. Asthma has been identified by WHO as one of the most significant non-communicable diseases in people of all ages and a major source of global economic burden, with the highest rates of asthma mortality occurring in LMICs [12]. Estimates of global childhood asthma prevalence come from the International Study of Asthma and Allergies in Childhood (ISAAC), which uses a standardized questionnaire for parent-reported history of wheeze [13]. Latin America, North America and Australia/New Zealand have the highest asthma prevalence among children 6–7 years (17–22%), but it is believed that there are high rates of undiagnosed asthma globally [14].

According to the Global Initiative for Asthma, asthma can be challenging to diagnose in children less than six years of age for the following reasons: (1) many young children experience viral-associated recurrent wheezing in the absence of asthma, and (2) measurements of airway obstruction using spirometry are challenging to perform in this age group and can be normal between symptomatic episodes [11], [15]. Global guidelines therefore recommend that asthma diagnosis in children less than six years of age be based on the presence of risk factors (e.g., family history of asthma/atopy, allergic sensitization) in combination with respiratory symptom patterns, response to therapeutic treatment trials, and the exclusion of alternate diagnoses [11]. As an alternative to spirometry, the forced oscillation technique (FOT) to measure respiratory system resistance and compliance has recently been shown to be a promising technique for the measurement of lung function in children as young as six weeks [16].

Asthma is believed to be caused by complex interactions between genes and the environment. Heritability estimates for asthma range from 25 to 95% and numerous markers of asthma risk have been identified, most notably polymorphisms at the chromosome 17q21 locus [17], [18]. Variable asthma prevalence among genetically similar populations living in different settings indicates that environmental influences are key in asthma development [19], [20], and some environmental risk factors for asthma appear to have the greatest effects in individuals with specific genetic risk variants [21], [22].

## 4 Biologic basis for an association between early life RSV LRTI and RWEC/asthma

An association between infant bronchiolitis and later development of asthma was first hypothesized in the late 1950 s [23]. Subsequent experimental studies have shown that mice infected with RSV have sustained airway hyperreactivity and histologic changes characteristic of human asthma that persist after clearance of the virus [24], and that early life RSV infection impairs regulatory T-cell function and increases susceptibility to allergic airway disease [25], [26]. In humans, increased RSV viral load [27] and disease severity [28], [29], [30] are associated with increased risk of RWEC and/or asthma in some studies but not in others [31], [32]. In one infant cohort, a distinct nasal immune response pattern to acute RSV illness was associated with increased risk of subsequent wheeze [33].

It is not well understood why some otherwise healthy infants develop severe LRTI when infected with RSV. Potential explanations include infection with a more virulent RSV strain (37–39), an aberrant host immune response [34], and/or the presence of other pre-existent determinants of vulnerability, both genetic and environmental (e.g. smoke exposure in utero and early life, crowding, and day care attendance). If pre-existent determinants of vulnerability cause severe disease with RSV infection, it is possible that they may also be independently predictive of an increased risk of developing RWEC and asthma in childhood. Evidence in support of this theory is provided by a prospective cohort study that assessed passive respiratory mechanics after birth, *prior* to any LRTI event, and found lower lung compliance and higher resistance to be associated with increased risk for both RSV hospitalization and number of days with subsequent wheeze in the first year of life [35]. Host genetic studies of RSV illness ascribe a genetic component to risk for severe infection [36] and several shared markers of risk for both RSV LRTI and asthma have been identified [17], [37], [38]. Twin studies also suggest a shared genetic risk for both diseases [39], [40], [41].

## 5 Evidence for an association between early life RSV LRTI and RWEC/asthma

### 5.1 Observational studies

Most of the evidence for an association between early life RSV LRTI and subsequent RWEC and asthma comes from observational studies, of which only two have been conducted in LMICs [42], [43]. These studies can be divided into two types: prospective studies that follow longitudinal cohorts of children forward in time, assessing them regularly for RSV LRTI and RWEC/asthma, and retrospective studies that use administrative databases to identify children who have had documented RSV LRTI and/or RWEC/asthma in the past.

The first type of prospective study is referred to here as a “medical event cohort study,” which defines exposure as an RSV LRTI inpatient or outpatient medical event, usually occurring within the first 1–2 years of life. Eligibility for enrollment into medical event cohort studies is therefore defined based on the known RSV LRTI exposure status. When studies compare this exposed group to those without RSV LRTI medical events, or to individuals hospitalized for a non-respiratory condition, many find a positive association between RSV LRTI and subsequent RWEC with odds ratios ranging from 3 to 36 [35], [37], [43], [44], [45], [46], [47], [48], [49], [50], [51], [52] and between RSV LRTI and asthma with odds ratios ranging from 3−17 [35], [42], [53], [54], [55], [56], [57], [58], [59], [60], [61]. In contrast, studies that compare individuals with RSV LRTI to those with LRTI due to other respiratory pathogens (e.g. human rhinovirus and bocavirus) usually find no difference in the risk of subsequent RWEC/asthma [29], [31], [62], [63], [64], [65], [66], [67], [68], [69], [70], [71], [72], [73], [74], or find RSV LRTI to be negatively associated with these outcomes compared to the non-RSV LRTI exposed [75], [76], [77], [78], [79], [80], [81], [82], [83], [84]. Several studies compared the same exposure group (with RSV LRTI medical events) to both types of comparison groups and found a positive association between RSV LRTI and RWEC/asthma when comparing exposed individuals to those without LRTI, but no significant association when compared to those with a non-RSV LRTI [37], [42], [53], [54], [76], [77], [85], [86], [87], [88], [89]. These studies suggest that LRTI due to some other respiratory viruses is as, or possibly more likely, to result in RWEC/asthma than RSV LRTI.

The second type of prospective study is a birth cohort study in which participants are enrolled in early infancy and prospectively surveilled for respiratory illnesses and RWEC/asthma outcomes. These include high-risk birth cohorts that enroll infants born preterm and/or with a family history of asthma or atopy [21], [90], [91], [92] as well as cohorts of healthy, term infants [93], [94], [95], [96]. Most compare children with RSV LRTI to those without LRTI of any type; some report positive associations with RWEC/asthma [91], [92], [93], [95], [97] and others find no association [21], [90], [94], [98]. Those that compare risk of RWEC/asthma in children with RSV LRTI compared to those with a non-RSV LRTI have found mixed results [96] or no difference in risk between LRTI groups with respect to future RWEC/asthma [99], [100].

A third type of prospective observational study follows non-randomized infants who received RSV mAbs [101], [102], [103], [104], [105], [106], [107], [108] or RSV immunoglobulin [103] based on clinical indications and compares RWEC and asthma outcomes in this group to children with similar clinical profiles who did not receive RSV immunoprophylaxis. Some of these studies showed a reduction in RWEC in preschool aged children but no effect on outcomes measured at older ages [101], [102], [106], one found a reduction in RWEC in nonatopic but not in atopic children [104], and others found no difference in asthma by treatment status [107], [108].

The association between RSV and RWEC/asthma can also be evaluated retrospectively, using administrative databases such as medical records. Administrative database studies have consistently shown associations between RSV LRTI hospitalization or unspecified bronchiolitis in early life and RWEC/asthma medical events in later life [32], [109], [110], [111], [112], [113], although only one such study required laboratory confirmation of RSV [111]. A study of children with primary RSV LRTI hospitalization before 24 months of age found that rates of subsequent asthma hospitalizations were approximately 4-fold higher in children first hospitalized with RSV LRTI between 6 and 24 months of age compared to children first hospitalized with RSV LRTI between 0 and 3 months of age [110]. A twin database in Denmark showed no difference in asthma or lung function among monozygotic twins discordant for RSV hospitalization in early life [39], [40], [41].

### 5.2 Randomized intervention studies

Two placebo-controlled randomized controlled trials (RCTs) of RSV mAbs have assessed RWEC and/or asthma outcomes. The first trial was an RCT of palivizumab conducted in healthy preterm Dutch infants that showed a decrease in the number of days with parent-reported wheezing in the first year of life and parent-reported current asthma at six years of age in the intervention group, but no difference in physician-diagnosed asthma or lung function at six years of age [114], [115]. The second trial was an RCT of motavizumab, an efficacious next generation mAb that ultimately was not pursued for licensure. The motavizumab trial was conducted in healthy, term Native American infants and found no difference between treatment groups in the incidence of medically attended wheezing between one and three years of age [116].

### 5.3 Systematic reviews of the available evidence

Several systematic reviews [37], [117], [118], [119] and two *meta*-analyses [120], [121] have assessed the evidence for an association between RSV illness and subsequent RWEC and/or asthma. The most recent systematic review without *meta*-analysis was published in 2017 as a part of a series of publications from the REGAL (RSV evidence – a Geographical Archival of the Literature) study. It included 74 publications from the United States, Canada, and Europe (including Turkey and the Russian Federation) [117]. Key findings were that early life RSV LRTI is strongly associated with RWEC and asthma persisting at least through early childhood, and with reduced lung function and increased airway reactivity. Preterm birth, Down syndrome and congenital heart disease were identified as potential effect modifiers that increase the strength of the association. A *meta*-analysis published in 2013 included 20 publications from 15 unique studies and found that children with RSV LRTI in early life had significantly higher relative odds of wheeze and asthma in later life compared to those without RSV LRTI (OR 3.84 [95%CI 3.23, 4.58]) [120]. A second *meta*-analysis, published in 2019, included 41 observational studies and excluded immunoprophylaxis studies [121]. It found that compared to children without respiratory symptoms in infancy, those with laboratory confirmed RSV illness in the first year of life had higher relative odds of RWEC through three years of age (OR 3.05 [95% CI 2.50.-3.71]) and between three and six years of age (OR 2.60 [95% CI 1.67–4.04]). Between six and twelve years of age, the relative odds of RWEC (OR 2.14 [95% CI 1.33–3.45]) and asthma (OR 2.95 [95% CI 1.96–4.46]) were both significantly greater in the RSV-exposed group. When the comparator group was infants with a non-RSV LRTI, there was no statistically significant association with subsequent RWEC or asthma for any of the age groups and when the comparator group was infants with human rhinovirus-associated LRTI, there was an inverse association with RWEC between three and six years of age (OR 0.41 [95% CI 0.20–0.83]). Finally, the WHO has commissioned a third systematic quantitative review and *meta*-analysis of epidemiologic and clinical trial data that will examine testable implications from both causal and non-causal models for the association between early life RSV LRTI and subsequent wheezing illness. A limitation of all *meta*-analyses on this topic is that it is challenging to compare results across studies given the use of different exposure and outcome definitions and underlying differences in the populations being studied.

## 6 Methodological considerations in defining a causal relationship between RSV LRTI and RWEC/Asthma

### 6.1 Observational studies

Selection bias, information bias, and confounding can each affect observational studies of RSV and RWEC/asthma. Selection bias can occur if children with severe RSV disease are more likely than those with less severe RSV LRTI to be enrolled and retained in a cohort through the study period. Information bias can occur via differential misclassification if children with a history of RSV LRTI are more prone to be diagnosed clinically with RWEC/asthma and/or undergo testing for asthma, or if children in the comparator group have RSV LRTI that is not detected. Misclassification bias can also be introduced if parents of children with RSV LRTI are more likely to report or remember wheezing episodes, and likewise, if parents of children with asthma more readily recall early RSV illness. Another potential source of misclassification bias is that many studies do not define a clear ‘washout’ period after the acute RSV illness, raising the possibility that some wheezing associated with the acute primary RSV disease episodes are misclassified as respiratory sequelae.

Confounding can be another source of bias in observational studies. Studies that do not adequately control for risk factors for both RSV LRTI and RWEC/asthma such as age, prematurity, access to health care, co-morbidities, exposure to indoor air pollution and secondhand smoke, and genetic susceptibility may be subject to a confounding bias that overestimates the association. Insufficient understanding of the shared genetic susceptibility for RSV LRTI, RWEC and asthma (e.g. specific immune markers or genes) limits the possibility to control for genetic confounding in observational designs. One approach to control for genetic confounding is to study twins. Although their statistical power is limited by their small size, studies of monozygotic twins discordant for RSV hospitalization in infancy have not shown evidence of differences in asthma prevalence or lung function [39], [40], [41]. Another approach is to capitalize on a quasi-random exposure variation, such as temporal variation in viral strain virulence, or periodic absences of circulating RSV. A specific example of this occurs annually due to the seasonal peaks of RSV circulation in temperate climates whereby children born just before the RSV season are at maximal risk for severe disease during their first few months of life when RSV circulation peaks. A study in Tennessee found birth four months before the winter virus peak to be associated with the highest risk for developing asthma [109]. Although less prone to confounding by a shared predisposition, birth timing studies can be confounded by other seasonal phenomena, such as non-RSV respiratory pathogens, allergens and other environmental exposures.

Another consideration in interpreting observational studies is the choice of comparison group. As noted earlier, a positive association between RSV LRTI and subsequent RWEC/asthma is consistently observed in studies that compare this exposure group to a comparator group without any LRTI medical event, but not when comparing to individuals with an LRTI caused by a pathogen other than RSV. This could be interpreted as meaning that multiple respiratory viruses are causal agents for RWEC/asthma, that LRTI itself is a causal agent, or that the susceptibility to develop LRTI when infected with any respiratory virus is a marker of underlying predisposition for RWEC/asthma.

Finally, although some non-randomized studies of RSV immunoprophylaxis in high-risk infants have found a reduction in RWEC or better lung function in treated compared to untreated infants [101], [102], [103], [105], [106], the absence of randomization makes these studies subject to biases. Moreover, the population risk profiles and the methods to evaluate the outcomes varied considerably in these studies, making it challenging to draw inferences across them [122]. Lastly, the restriction to high-risk infants with a clinical indication for immunoprophylaxis limits the ability to generalize their results to the general infant population.

### 6.2 Randomized controlled trials of monoclonal antibodies

The greatest advantage of RCTs is that confounding by a shared predisposition for both the exposure and outcome should be eliminated. However, RCTs can be subject to misclassification bias, particularly if unmasking of the treatment assignment occurs before the end of follow up. There may have been such bias in the Dutch palivizumab RCT that showed a decrease in parent-reported asthma at six years of age after unmasking had occurred, but no difference in more objective measures including physician-diagnosed asthma or lung function [114].

A limitation of RCTs of RSV mAbs and vaccines is that they require very large sample sizes to detect an association with most RWEC/asthma outcomes. A recent analysis used systematic reviews and expert opinions to test 81 sample size assumption scenarios, with risk ratios between maternal vaccination and recurrent wheezing ranging from 0.9 to 1.0 for 70% of the scenarios [3]. Scenarios were ranked according to plausibility, with 75% of plausible scenarios requiring a sample size greater than 30,000 and 47% requiring a sample size greater than 100,000 mother-infants per trial arm. According to this analysis, the two mAb RCTs described above, as well as a recently completed phase III maternal RSV vaccine trial (ClinicalTrials.gov ID: NCT02624947), would have been underpowered to find a statistically significant effect on RWEC and asthma.

## 7 Recommendations for future studies

This report summarizes many of the methodologic challenges faced by studies that aim to assess (1) whether there is a causal association between early life RSV LRTI and subsequent RWEC and asthma, or (2) whether an effective RSV preventive product could be expected to reduce the risk of subsequent RWEC/asthma. Recognizing these limitations, the participants discussed good practices for designing and analyzing future studies in order to maximize their contribution to the evidence base. This guidance is presented in Table 1A, Table 1B and summarized below:

**Table 1A:** Study designs to assess a causal association between early life RSV lower respiratory tract infection and subsequent recurrent wheeze of early childhood and asthma.

Design	Time required to conduct study	Resources required	Sample size required	Feasible in LMIC^1^ setting?	Strengths	Limitations	Guidance for future studies
Prospective longitudinal cohort study (event-based or birth cohort)	Long	Medium to high	Medium to large	Yes	▪ Can capture most exposure events ▪ Can measure outcomes longitudinally ▪ Can measure co-variates of interest prospectively	▪ Observational, non-randomized ▪ Subject to biases ▪ Common predisposition (e.g., genetic confounder) cannot be ruled out ▪ Loss to follow-up ▪ Choice of comparison group can affect results (e.g., no LRTI vs. non-RSV LRTI)	Additional studies using this design offer limited potential for further insight and should only be done (1) if improved measurements of shared predisposition can be measured (e.g., genetic markers), (2) if they assess quasi-random exposures to RSV LRTI (e.g., birth timing) or (3) if lung function is measured *before* first RSV exposure
Retrospective cohort studies using administrative data	Short	Low to medium	Large	No	▪ Large sample size available ▪ Can evaluate subgroups of interest and effect modification ▪ Can be done more quickly and with fewer resources compared to most other designs	▪ Observational, non-randomized ▪ Imprecise definitions of exposure and outcome are possible ▪ Subject to biases ▪ Some co-variates of interest may not be available	Additional studies using this design offer limited potential for further insight and should be limited to studies that can incorporate birth timing to reduce bias in the exposure variable.
Randomized controlled trials or vaccine probe studies	Long	High	Large	Yes	▪ Randomized exposure ▪ Standardized exposure and outcome measurements make meta analyses possible ▪ Can measure co-variates of interest prospectively	▪ Very large sample size required ▪ Requires several years of follow up ▪ RSV LRTI protection period may be limited to a few months (in the case of maternal vaccines and mAbs) ▪ Definitions may be difficult to standardize in practice across different settings ▪ Potential loss to follow up	This design has greater potential to establish causal association than observational studies. Individual studies should be powered to assess an RWEC/asthma outcome. If not possible, standardized assessments should be used so that data from multiple RCTs can be pooled for analysis. An absence of effect does not establish that there is not a causal relationship. Vaccination allocation should remain masked until the end of long-term follow-up. If this is not possible, a priority should be placed on objective measurement of outcomes with blinded analysis.

1 Low and middle-income countries

**Table 1B:** Study designs to assess whether RSV vaccines and monoclonal antibodies can reduce risk of recurrent wheeze of early childhood and asthma.

Design	Time required to conduct study	Resources required	Sample size required	Feasible in LMIC^1^ setting?	Strengths	Limitations	Guidance for future studies
Randomized controlled trials or vaccine probe studies	Long	High	Large	Yes	▪ Randomized exposure ▪ Standardized exposure and outcome measurements make meta analyses possible ▪ Can measure co-variates of interest prospectively	▪ Very large sample size required ▪ Requires several years of follow up ▪ RSV LRTI protection period may be limited to a few months (in the case of maternal vaccines and monoclonal antibodies) ▪ Definitions may be difficult to standardize in practice across different settings ▪ Potential loss to follow up	Acceptable, with requirement for standardized definitions to allow for *meta*-analyses, and with caveat that most individual trials will be underpowered to find an association. Vaccination allocation should remain masked until the end of long-term follow-up
Post introduction case-control study	Short^2^	Medium	Small- medium	Yes	▪ Relatively quick to conduct ▪ Smaller sample size needed	▪ Prone to bias and confounding, particularly for multi-cause syndromes like asthma ▪ Shared predisposition cannot be ruled out ▪ Vaccination histories difficult to reliably obtain retrospectively	Not recommended in most settings due to high risk of confounding and bias.
Post introduction pre-post impact study ▪ Post introduction administrative database study	Long	High	Large	Only if surveillance like DSS established before introduction	▪ Large sample sizes are potentially available ▪ Not subject to selection bias	▪ Ecological fallacy possible – temporal trends can influence hospitalization and asthma rates ▪ Impact cannot be observed until years after introduction ▪ Pre-vaccination incidence must be established over several years	Not recommended in most settings due to unclear temporal trends in asthma prevalence. It is unknown whether recurrent wheeze of early childhood is also subject to such time-dependent variability.
Phased introduction	Long	High	Large	Yes	▪ Provides for a contemporaneous comparison group ▪ Could be group randomized	▪ Comparison areas/populations could differ in terms of temporal trends and other confounding factors, leading to bias ▪ Not feasible everywhere due to policy constraints ▪ Impact cannot be observed until years after introduction ▪ Potential for movement between introduction areas resulting in contamination of groups	Acceptable, if appropriate surveillance is in place and if potential confounders can be identified and adequately controlled for.

1 Low and middle-income countries

2 A short amount of time is needed to accrue participants in case control studies, but recurrent wheeze and asthma outcomes cannot be assessed until several years after vaccination.

*Observational studies:* Additional observational studies using conventional designs were considered to be of little value in further elucidating the causal link between RSV LRTI and RWEC/asthma, with a few exceptions. Observational studies that would be of value are those that incorporate measures of neonatal immune function or pre-exposure lung function assessments, and those that involve quasi-random exposure to RSV in specific geographical settings.

*Randomized controlled trials:* RCTs were considered to be the least biased study design to assess both the questions of causal association and whether RSV preventive products can reduce subsequent RWEC/asthma, but they require investment in sufficiently powered individual trials and/or the use of standardized measures of exposure and outcome to allow pooling of data across multiple studies for meta-analyses.

*Post-introduction studies:* Given the large sample sizes required by RCTs, post-introduction studies conducted after RSV vaccines/mAbs are licensed and introduced into national programs were considered to be promising strategies to address these questions. Examples include pre-post ecological studies, case-control studies, and phased introduction studies. Pre-post studies, where population-level rates of RWEC/asthma before and after vaccine introduction are compared, offer a straightforward approach but are not recommended to address these questions due to important limitations. In addition to requiring high quality pre-introduction surveillance data, they are susceptible to bias due to temporal trends in disease prevalence. This is a particular risk for asthma outcomes because asthma prevalence is not constant within communities over time and secular trends in risk factors such as diet, antibiotic use, urbanization and air pollution can be difficult to control for [123]. Case-control studies that compare vaccination status in children with and without the outcome of interest are commonly used to evaluate vaccine effectiveness post-introduction. However, such case-control studies are often biased in that unvaccinated children differ from vaccinated children in ways that are related to the outcome of interest; in this case their propensity to be diagnosed with RWEC/asthma. Therefore, case-control studies to answer this question were not considered to be appropriate. Phased introduction, whereby a vaccine is sequentially introduced to defined geographic areas, offers the most promising design to address whether RSV preventive products can reduce the risk of subsequent RWEC/asthma. By comparing contemporaneous cohorts of RSV-vaccinated and unvaccinated children, phased introduction addresses year-to-year variability and minimizes confounding by temporal factors. Like pre-post studies, it requires a robust surveillance system to be in place prior to vaccine introduction and to be maintained throughout the follow-up period. It also requires that populations with early access to the vaccine do not differ in important ways from populations with delayed access to the vaccine (including with respect to exposure to environmental risk factors, such as air pollution), and that outcome ascertainment does not differ by introduction group. In some situations, the areas for vaccine introduction can be randomly assigned. Examples of this are WHO’s pilot programme for the RTS, S/AS01 malaria vaccine [124], the phased introductions of PCV in Mongolia [125], and hepatitis B vaccine in The Gambia [126].

Given the limitations of each approach, a combined strategy incorporating evidence from long-term follow up of randomized trials in addition to post-introduction data will likely be required to determine whether vaccines and mAbs reduce RWEC/asthma. A challenge of all prospective study designs is retaining participants throughout the 3–5 years of follow up that are required before outcomes can be assessed. Regardless of design, all studies conducting long-term follow up should assess the comparability of those who remain in the study to those who are lost to follow-up.

Finally, the meeting participants identified key variables, definitions and measurements that future studies assessing these questions should consider (Table 2). The participants recommended that the primary exposure of interest should be laboratory-confirmed RSV LRTI between birth and two years. Guidance for defining the exposure was aligned with advice from a previous WHO consultation that recommended using the Integrated Management of Childhood Illness (IMCI) definitions of LRTI [127], with inclusion of objective measures of severity such as tachypnea and oxygen saturation [128].

**Table 2:** Key variables, definitions and measurements for future studies of the association between RSV lower respiratory tract infection and subsequent recurrent wheeze of early childhood (RWEC) and asthma.

**Defining the exposure:**	• *Exposure period* o Between birth and two years, may vary by study design • *Microbiological confirmation*: o Assays that allow for identification of RSV viral strains (A/B) are optimal o Multiplex PCR assays should be used to identify co-infecting respiratory pathogens, when possible o RSV gene sequencing and RSV serology at 12 months of age in conjunction with methods above are lower priority but can be considered along with the other diagnostic methods • *Definition of lower respiratory tract infection (LRTI)***:** o The LRTI clinical case definition should be based on Integrated Management of Childhood Illness (IMCI) criteria o Both LRTI inpatient and outpatient events should be included since hospitalization criteria can vary widely by study setting • *Measures of severity*: o The following should be collected: respiratory rate, oxygen saturation, temperature, auscultation, cough, subcostal retractions, and difficulty breast feeding/feeding o Quantitative measures should be recorded using a continuous scale to allow for flexibility in categorization that can be compared across settings o A combination of these variables can be used to generate severity scores that can be compared across settings

**Defining the outcome:**	• *Measuring RWEC and asthma* o Objective measures should be prioritized, including medically attended outcomes and lung function testing o Parent/caregiver reports can provide useful supplemental information when standardized assessments are used; examples of Standardized Definitions include the 2019 Brighton Collaboration definitions for acute wheeze in the pediatric population. o In randomized trials, caregivers and physicians should be masked to treatment group allocation o Continuous outcomes (e.g. number of medically attended wheezing events) should be reported whenever possible. In LMIC^1^ settings with low literacy, phone calls are recommended over diaries. Audio and video clips can be used to standardize reporting o Medical costs and burden on the health system, absences from work and school, can be useful to collect depending on the setting • *Measuring lung function* o Forced oscillation technique with bronchodilation is more sensitive than spirometry for the detection of abnormal resistance, can be used in young children, and can be done in the field in LMIC settings • *Follow up period* o RWEC outcomes should be reported annually for each year of life, with follow up until at least three years of age o Asthma outcomes should be assessed at six years of age or later
**Potential confounders and effect modifiers to measure**	• *High priority co-variates of interest* o Birth weight, which can be a proxy for compromised lung function and development at birth o Preterm birth, which is associated with both RSV LRTI and RWEC/asthma, but can be difficult to ascertain in LMICs o Family history of asthma/atopy • *Additional co-variates of interest* o Co-infections with other respiratory pathogens o Other medically attended LRTIs o Vaccination status o Sex o Ethnic group o Timing of birth relative to the RSV season o Age at the time of first RSV LRTI illness o Smoke exposure (including maternal smoking during pregnancy, household smoking after birth, and ambient air pollution) o Mode of delivery (vaginal vs. caesarean section) o Access to health care o Vaccination status o Household crowding index o Nutritional status
**Subgroups of interest**	• Infants born preterm, with down syndrome or congenital heart disease

There was agreement that the primary long-term outcomes of early life RSV LRTI that are of public health interest are RWEC, measured until at least three years of age, and asthma, measured at six years of age or later, and that studies should prioritize medically attended outcomes using standard definitions. FOT is a promising tool for objective measures of lung function in infants and young children and can be considered for use in all settings, including LMICs [16]. In clinical trials, study personnel should remain masked to treatment allocation for the entire duration of follow up to minimize bias in the follow up of long-term outcomes, particularly since infants will have passed the critical age for immunization once the trial has ended. Objective measures of outcomes with blinded analysis should be prioritized.

Potential confounders are important to measure in observational studies to the extent possible but some, such as genetic susceptibility, are very difficult to control for. Simple, standardized data collection methods for all co-variates of interest are preferred, with birth weight, preterm birth, and family history of asthma and atopy identified as the highest priority. Finally, although studies are unlikely to be powered to detect effect modification, information about preterm birth, Down syndrome, and congenital heart disease should be collected if available.

## 8 Policy considerations

The meeting participants agreed that, given the current knowledge of the potential public health benefit, RSV vaccine policy decisions should be based on the efficacy and impact against the spectrum of acute illness caused by RSV LRTI in infants and young children, with the primary focus being prevention of severe disease. Definitive data on the impact of RSV vaccines/mAbs on subsequent RWEC and asthma are unlikely to be available at the time vaccine policy recommendations are made. If high-quality, robust evidence does eventually support a preventive role of RSV vaccines/mAbs for RWEC/asthma, the additional longer term health and economic benefits related to RWEC/asthma prevention could contribute to policy-making in some countries.

## 9 Summary and conclusions

This WHO-sponsored meeting was convened to evaluate the current evidence for a causal association between RSV LRTI in young children and subsequent development of RWEC/asthma, to assess the potential for RSV vaccines and mAbs to reduce the risk of RWEC and asthma, and to provide guidance for future studies that are poised to address these questions. The evaluation of the evidence was focused on the body of epidemiological literature rather than the experimental data from animals and humans. Moreover, the application of causal modelling techniques to the epidemiologic data were not considered, but will be addressed in the forthcoming WHO commissioned systematic review and *meta*-analysis [129]. The meeting participants concluded that most observational studies show an association between RSV LRTI and RWEC and asthma; however, the interpretation of these studies, as they were performed, is subject to potential measured and unmeasured biases. The most compelling counter-argument against a causal association is that there could be a shared predisposition for both severe RSV disease and RWEC/asthma and that having severe disease with an RSV infection is a marker of this predisposition. RCTs of RSV mAbs did not show efficacy against objective measures of RWEC/asthma, although they were not powered to do so.

After reviewing the evidence, the participants resolved that: (i) the current epidemiological evidence is inconclusive in establishing a *causal* association between RSV LRTI and RWEC/asthma, (ii) the evidence does not establish that RSV mAbs and vaccines are likely to have a substantial effect on these outcomes and (iii) the prevention of severe, acute RSV disease in young children, a well-established, substantial public health burden, should continue to be the highest priority for policy-setting bodies deliberating on RSV vaccine and mAb recommendations, regardless of their impact on subsequent RWEC and asthma (Panel 1). RSV vaccine impact and economic models should limit prevention of RWEC/asthma to sensitivity analyses, and RSV vaccine policy decisions should not include impact on RWEC/asthma prevention.

**Panel 1:** Key points on the causal association between RSV lower respiratory tract infection and subsequent recurrent wheeze of early childhood (RWEC) and asthma.

*RSV disease in young children* ▪ The burden of RSV infection in young children is high, with almost all children having been exposed by age 2 years. Severe RSV illness represents a sizeable minority of all RSV infections (15–50%). ▪ The prevention of severe RSV disease in young children is the primary outcome of RSV-illness prevention from a public health perspective, regardless of the causal association with RWEC/Asthma. *RWEC and asthma* ▪ RWEC is common, occurring in approximately one-fifth of children. The mean global estimate of asthma prevalence at age 6–7 is approximately 11%, with wide variation by region. ▪ RWEC/Asthma prevalence and determinants are better understood in high income countries than low and middle income countries (LMICs). More data are needed in LMICs to better understand the burden. *Association between RSV-LRTI and RWEC/asthma* ▪ RSV-LRTI in infancy is associated with the later development of RWEC/asthma, though it is not known whether the association is causal. ▪ Severe RSV infection with lower respiratory tract involvement is more strongly associated with the development of RWEC/asthma than non-severe RSV infection. ▪ RWEC and asthma are complex conditions with multiple phenotypes, and likely multiple individual and overlapping etiologies. Therefore, the fraction of these outcomes that is potentially preventable by RSV vaccines/mAbs is likely to be modest, but may vary by population. *Causal association between RSV-LRTI and RWEC/asthma* ▪ Epidemiologic studies and clinical trials present mixed evidence for a *causal* association between RSV infection and RWEC/asthma, which might in part be due to different study designs, methodologies, and study populations. ▪ The state of current evidence is inconclusive in establishing a causal association between RSV infection and RWEC/asthma. ▪ RSV vaccine impact and economic models should limit prevention of RWEC/asthma to sensitivity analyses, and RSV vaccine policy decisions should not include impacts on RWEC/asthma prevention. ▪ Additional high-quality evidence addressing the question of the potential for RSV vaccines/mAbs to prevent RWEC/asthma would be valuable. Such studies should follow good practice guidance with respect to study design and the use of standardized measurements and definitions across diverse settings.

Nonetheless, the participants considered that the high burden of RWEC and asthma justifies the continued study of the association between these two conditions, and that a better understanding of the association could contribute to establishing the public health value of RSV vaccines and mAbs. Regardless of whether a causal association exists, the burden of RWEC/asthma in LMICs needs to be elucidated and benchmarked to other public health priorities. Future epidemiological studies that examine the association should follow good practice guidance (Table 1A, Table 1B) using standardized methods to collect and define key variables (Table 2). RCTs of RSV vaccines and mAbs provide the best opportunity to probe whether a causal association exists in an unbiased way, and such studies may consider long-term follow-up of participants to measure RWEC, and if possible, asthma, using standardized methods to allow for pooled analysis. Moreover, eventual large-scale introduction of RSV preventive products might create opportunities to assess the causal association between RSV and RWEC/asthma at a population level. The design of post-introduction evaluations and the development of baseline surveillance platforms should be considered prior to introductions, particularly in LMICs where data on the burden of RWEC/asthma are limited.

Both RSV associated LRTI and RWEC/asthma confer a substantial disease burden in children globally. To identify a single intervention, such an RSV vaccine or mAb, that lessens the burden of both diseases would be a fortuitous public health success. Efforts should continue to better understand whether this can be achieved. Nonetheless, lack of conclusive evidence for a dual preventive impact should not slow the pursuit of new preventive approaches independently targeting each of these important diseases of childhood.

## Declaration of Competing Interest

The authors declare the following financial interests/personal relationships which may be considered as potential competing interests: LB has regular interaction with pharmaceutical and other industrial partners. He has not received personal fees or other personal benefits. He is the founding chairman of the ReSViNET Foundation. JAE has served as a consultant to Sanofi Pasteur and Meissa Vaccines and her institution receives support from Novavax, AstraZeneca, Merck, and GlaxoSmithKline. LLH reports research grants to her institution from Novavax, Merck, GSK, and Pfizer. TVH receives funding relevant to the submitted work from the National Institutes of Health and the WHO and served on the Pfizer RSV Infant/Maternal Health External Advisory Board in 2019. HN has received grant funding from Bill and Melinda Gates Foundation, Sanofi Pasteur, World Health Organization and Innovative Medicines Initiative. HN has received honoraria and speaker fees from Sanofi Pasteur, Abbvie and Janssen. EAFS reports grant support and personal fees in the last 36 months from Astra Zeneca Inc., Merck & Co., Regeneron Inc., and Pfizer Inc.; grant support from Novavax; and personal fees from Roche Inc. AbbVie Inc., and Alere Inc.

## Figures and Tables

**Table:** 

Session	Presenter	Objectives
1. **Opening**		
Welcome	Martin Friede	Welcome from Director, Initiative Vaccine Research, IVB, WHO
Overview and meeting objectives	Daniel Feikin	Introduction of participants. Overview of meeting
2. **RSV, early childhood wheeze and asthma: background**		
RSV 101 – RSV infections in young infants	Jan Englund	Describe spectrum of RSV illness in infants. Provide basis for case definition discussions..
Asthma and wheeze 101 – Epidemiology and causes of asthma and recurrent wheeze in early childhood (RWEC); Biological basis of the RSV-wheeze association	Tina Hartert	Describe epidemiology and clinical basis of recurrent wheeze in early childhood and asthma. Distinguish from acute wheeze with RSV. Describe potential mechanisms for causative association with RSV illness. Describe genetic predisposition for severe RSV disease and asthma.
Measures of wheeze and asthma in vaccine clinical trials	Heather Zar	Discuss measures of asthma and recurrent wheeze in early childhood. Discuss sens/spec of different clinical trial endpoints. Basis for discussion of outcome definitions
3. **Evidence for/against causal association between RSV and recurrent wheeze/asthma?**		
Observational studies: Long-term respiratory morbidity associated with RSV in early childhood	Eric Simoes	Provide overview of the REGAL systematic review; highlight seminal longitudinal cohort studies.
RCTs I: Palivizumab (Dutch MAKI trial) and II: Motavizumab in healthy Native American Infants	Nienke Scheltema & Laura Hammitt	Review findings from these two RCTs and describe ongoing motavizumab participant follow up.
Use of administrative datasets	Deshayne Fell	Use of administrative databases to evaluate the RSV - RWEC/Asthma association
BMGF Perspective	Prachi Vora	Present BMGF perspective on importance of understanding RSV/RWEC/asthma association
Critical Review of Evidence and Applied Methodology	Steven Brunwasser	To present results of the RSV/RWEC/Asthma critical review
4. **Methodological Issues**		
Potential biases in observational studies	David Savitz	Discuss biases in observational studies
Sample size analysis RCTs of maternal RSV vaccines	Justin Ortiz	Results of modelling exercise of sample size needed to detect true association of RSV and RWEC/asthma
Post introduction Study Design Considerations	Kim Mulholland	Present different study design options to assess long-term outcomes post introduction RSV vaccine/mAb (phase IV)
5. **Questions for Recommendation – Part 1**		
Strategic questions for recommendation	Daniel Feikin	Describe process for tackling strategic questions
Small group break-out sessions	All	Groups to break out to discuss assigned questions

**Table:** 

Session	Presenter	Objectives
Recap of Day 1, Objectives for Day 2	Daniel Feikin	
6. **Potential policy Implications of the RSV/ERCW/Asthma association**		
Advisory Committee Perspective – A panel discussion	Ruth Karron, Fred Were, Kate O’Brien	Discuss how RWEC/asthma could relate to advisory group deliberations on RSV vaccines
Long-term follow-up of Novavax vaccine	Heather Zar	Plans for long term follow-up of Novavax trial participants
7. **Questions for recommendation – Part 2**		
Small groups reconvene		Finalize recommendations
Small groups presentation (1–2)	All	Small groups present conclusions
Small groups – continued (3–4)	All	Small groups present conclusions
Editorial review of evidence presented – how to think about causation?	Peter Smith	Establish framework for determining causation
Large group discussion –study design	All	Group to discuss and weigh what the best practice study designs
Group Statement on state of the evidence	All	Group to develop a statement assessing the state of the evidence that RSV is causally related to RWEC/asthma
Closing remarks	Daniel Feikin	

## AGENDA

### Organizer: Daniel Feikin, WHO

Chair: Bruce Innis, PATH

Rapporteur: Amanda Driscoll, Univ. Maryland

Day 1

#### PARTICIPANTS

1 **Syed Hasan Arshad**, David Hide Asthma and Allergy Centre and University of Southampton, UK
2 **Louis Bont**, University Medical Centre Utrecht, the Netherlands
3 **Steven Brunwasser**, Department of Medicine, Vanderbilt University School of Medicine, Nashville, USA
4 **Thomas Cherian**, Independent, Chairman IVR Technical Advisory Group on RSV Vaccines, Geneva, Switzerland
5 **Amanda Driscoll**, Center for Vaccine Development and Global Health, University of Maryland School of Medicine, Baltimore, USA
6 **Jan Englund**, Department of Pediatrics, University of Washington School of Medicine, Seattle, USA
7 **Deshayne Fell**, School of Epidemiology and Public Health, University of Ottawa, Ottawa, Canada
8 **Daniel Feikin**, Initiative for Vaccine Research, Department of Immunizations, Vaccines and Biologicals, World Health Organization
9 **Tina Hartert**, Center for Asthma Research, Department of Medicine, Vanderbilt Institute for Medicine & Public Health, Vanderbilt University Medical Center, Nashville, USA
10 **Bruce Innis**, PATH, Respiratory Infections and Maternal Immunizations, PATH Center for Vaccine Innovation and Access, Washington DC, USA
11 **Ruth Karron**, Center for Immunization Research, Johns Hopkins Vaccine Initiative, Bloomberg School of Public Health, Johns Hopkins University, Baltimore, USA
12 **Gayle Langley**, Division of Viral Diseases, Respiratory Viruses Branch, Centers for Disease Control and Prevention, Atlanta, USA
13 **Kim Mulholland**, Murdoch Childrens’ Research Institute, Melbourne, Australia
14 **Harish Nair**, Paediatric Infectious Diseases and Global Health, Centre for Global Health Research, University of Edinburgh, Edinburgh, UK
15 **Patrick Munywoki**, CDC-Kenya, Nairobi, Kenya
16 **Laura Hammitt**, International Vaccine Access Center, Johns Hopkins Vaccine Initiative, Center for American Indian Health, Johns Hopkins Bloomberg School of Public Health, Baltimore, USA
17 **Justin Ortiz**, Center for Vaccine Development, University of Maryland School of Medicine, Baltimore, USA
18 **David Savitz**, Brown University, Providence, Rhode Island, USA
19 **Nienke Scheltema**, University Medical Centre Utrecht, The Netherlands
20 **Eric Simoes**, University of Colorado, Denver, USA
21 **Peter Smith**, London School of Hygiene and Tropical Medicine, London, UK
22 **Fred Were**, School of Medicine, University of Nairobi, Kenya < frednwere@gmail.com>
23 **Heather Zar**, Department of Paediatrics and Child Health, University of Cape Town, South Africa

## OBSERVERS

1 **Prachi Vora**, Associate Program Officer, Global Health, Bill and Melinda Gates Foundation, Seattle, USA
2 **Deborah Higgins**, Director, RSV Vaccine Project, PATH, Seattle, USA
