# Supplementary material for: Does respiratory syncytial virus lower respiratory illness in early life cause recurrent wheeze of early childhood and asthma? Critical review of the evidence and guidance for future studies from a World Health Organization-sponsored meeting
Source: Vaccine. 2020 Mar 4;38(11):2435–48. doi: 10.1016/j.vaccine.2020.01.020 (PMC7049900; doi:10.1016/j.vaccine.2020.01.020)
Supplement: Supplementary data 1 [file mmc1.docx]

**AGENDA**

***Organizer: Daniel Feikin, WHO***

***Chair: Bruce Innis, PATH***

***Rapporteur: Amanda Driscoll, Univ. Maryland***

**Day 1**

| **Session** | **Presenter** | **Objectives** |
| --- | --- | --- |
| 1. **Opening** | | |
| Welcome | Martin Friede | Welcome from Director, Initiative Vaccine Research, IVB, WHO |
| Overview and meeting objectives | Daniel Feikin | Introduction of participants. Overview of meeting |
| 1. **RSV, early childhood wheeze and asthma: background** | | |
| RSV 101 – RSV infections in young infants | Jan Englund | Describe spectrum of RSV illness in infants. Provide basis for case definition discussions.  . |
| Asthma and wheeze 101 – Epidemiology and causes of asthma and recurrent wheeze in early childhood (RWEC); Biological basis of the RSV-wheeze association | Tina Hartert | Describe epidemiology and clinical basis of recurrent wheeze in early childhood and asthma. Distinguish from acute wheeze with RSV. Describe potential mechanisms for causative association with RSV illness. Describe genetic predisposition for severe RSV disease and asthma. |
| Measures of wheeze and asthma in vaccine clinical trials | Heather Zar | Discuss measures of asthma and recurrent wheeze in early childhood. Discuss sens/spec of different clinical trial endpoints. Basis for discussion of outcome definitions |
| 1. **Evidence for/against causal association between RSV and recurrent wheeze/asthma?** | | |
| Observational studies: Long-term respiratory morbidity associated with RSV in early childhood | Eric Simoes | Provide overview of the REGAL systematic review; highlight seminal longitudinal cohort studies. |
| RCTs I: Palivizumab (Dutch MAKI trial) and II: Motavizumab in healthy Native American Infants | Nienke Scheltema & Laura Hammitt | Review findings from these two RCTs and describe ongoing motavizumab participant follow up. |
| Use of administrative datasets | Deshayne Fell | Use of administrative databases to evaluate the RSV - RWEC/Asthma association |
| BMGF Perspective | Prachi Vora | Present BMGF perspective on importance of understanding RSV/RWEC/asthma association |
| Critical Review of Evidence and Applied Methodology | Steven Brunwasser | To present results of the RSV/RWEC/Asthma critical review |
| 1. **Methodological Issues** | | |
| Potential biases in observational studies | David Savitz | Discuss biases in observational studies |
| Sample size analysis RCTs of maternal RSV vaccines | Justin Ortiz | Results of modelling exercise of sample size needed to detect true association of RSV and RWEC/asthma |
| Post introduction Study Design Considerations | Kim Mulholland | Present different study design options to assess long-term outcomes post introduction RSV vaccine/mAb (phase IV) |
| 1. **Questions for Recommendation – Part 1** | | |
| Strategic questions for recommendation | Daniel Feikin | Describe process for tackling strategic questions |
| Small group break-out sessions | All | Groups to break out to discuss assigned questions |

**Day 2**

| **Session** | **Presenter** | **Objectives** |
| --- | --- | --- |
| Recap of Day 1, Objectives for Day 2 | Daniel Feikin |  |
| 1. **Potential policy Implications of the RSV/ERCW/Asthma association** | | |
| Advisory Committee Perspective – A panel discussion | Ruth Karron, Fred Were, Kate O’Brien | Discuss how RWEC/asthma could relate to advisory group deliberations on RSV vaccines |
| Long-term follow-up of Novavax vaccine | Heather Zar | Plans for long term follow-up of Novavax trial participants |
| 1. **Questions for recommendation – Part 2** | | |
| Small groups reconvene |  | Finalize recommendations |
| Small groups presentation (1-2) | All | Small groups present conclusions |
| Small groups – continued (3-4) | All | Small groups present conclusions |
| Editorial review of evidence presented – how to think about causation? | Peter Smith | Establish framework for determining causation |
| Large group discussion –study design | All | Group to discuss and weigh what the best practice study designs |
| Group Statement on state of the evidence | All | Group to develop a statement assessing the state of the evidence that RSV is causally related to RWEC/asthma |
| Closing remarks | Daniel Feikin |  |

**PARTICIPANTS**

1. **Syed Hasan Arshad**, David Hide Asthma and Allergy Centre and University of Southampton, UK <S.H.Arshad@soton.ac.uk>
2. **Louis Bont**, University Medical Centre Utrecht, the Netherlands <L.Bont@umcutrecht.nl>
3. **Steven Brunwasser**, Department of Medicine, Vanderbilt University School of Medicine, Nashville, USA Steven.brunwasser@vumc.org
4. **Thomas Cherian**, Independent, Chairman IVR Technical Advisory Group on RSV Vaccines, Geneva, Switzerland. <cheriant@mmglobalhealth.org>
5. **Amanda Driscoll**, Center for Vaccine Development and Global Health, University of Maryland School of Medicine, Baltimore, USA <ADriscoll@som.umaryland.edu>
6. **Jan Englund**, Department of Pediatrics, University of Washington School of Medicine, Seattle, USA janet.englund@seattlechildrens.org
7. **Deshayne Fell**, School of Epidemiology and Public Health, University of Ottawa, Ottawa, Canada DFell@cheo.on.ca
8. **Daniel Feikin**, Initiative for Vaccine Research, World Health Organization feikind@who.int
9. **Tina Hartert**, Center for Asthma Research, Department of Medicine, Vanderbilt Institute for Medicine & Public Health, Vanderbilt University Medical Center, Nashville, USA <tina.hartert@vumc.org>
10. **Bruce Innis**, PATH, Respiratory Infections and Maternal Immunizations, PATH Center for Vaccine Innovation and Access, Washington DC, USA <binnis@path.org>
11. **Ruth Karron**, Center for Immunization Research, Johns Hopkins Vaccine Initiative, Bloomberg School of Public Health, Johns Hopkins University, Baltimore, USA rkarron@jhu.edu
12. **Gayle Langley**, Division of Viral Diseases, Respiratory Viruses Branch, Centers for Disease Control and Prevention, Atlanta, USA <fez7@cdc.gov>
13. **Kim Mulholland**, Murdoch Childrens’ Research Institute, Melbourne, Australia <Kim.Mulholland@lshtm.ac.uk>
14. **Harish Nair**, Paediatric Infectious Diseases and Global Health, Centre for Global Health Research, University of Edinburgh, Edinburgh, UK <Harish.Nair@ed.ac.uk>
15. **Patrick Munywoki**, CDC-Kenya, Nairobi, Kenya oha6@cdc.gov
16. **Laura Hammitt**, International Vaccine Access Center, Johns Hopkins Vaccine Initiative, Bloomberg School of Public Health, Johns Hopkins University, Baltimore, USA lhammitt@jhu.edu
17. **Justin Ortiz**, Center for Vaccine Development, University of Maryland School of Medicine, Baltimore, USA <JOrtiz@som.umaryland.edu>
18. **David Savitz**, Brown University, Providence, Rhode Island, USA <david_savitz@brown.edu>
19. **Nienke Scheltema**, University Medical Centre Utrecht, The Netherlands <n.m.scheltema@umcutrecht.nl>
20. **Eric Simoes**, University of Colorado, Denver, USA <Eric.Simoes@ucdenver.edu>
21. **Peter Smith**, London School of Hygiene and Tropical Medicine, London, UK peter.smith@lshtm.ac.uk
22. **Fred Were**, School of Medicine, University of Nairobi, Kenya <frednwere@gmail.com>
23. **Heather Zar**, Department of Paediatrics and Child Health, University of Cape Town, South Africa <Heather.Zar@uct.ac.za>

**OBSERVERS**

1. **Prachi Vora**, Associate Program Officer, Global Health, Bill and Melinda Gates Foundation, Seattle, USA Prachi.Vora@gatesfoundation.org
2. **Deborah Higgins**, Director, RSV Vaccine Project, PATH, Seattle, USA <dhiggins@path.org>
